# Supplementary material for: Fluorescent Aromatic Polyether Sulfones: Processable, Scalable, Efficient, and Stable Polymer Emitters and Their Single-Layer Polymer Light-Emitting Diodes
Source: Nanomaterials (Basel). 2024 Jul 25;14(15):1246. doi: 10.3390/nano14151246 (PMC11313940; doi:10.3390/nano14151246)
Supplement: Supplementary file 1 [file nanomaterials-14-01246-s001.zip › nanomaterials-3055469-supplementary.pdf]

Supporting Information

Fluorescent Aromatic Polyethersulfones: Processable, Scalable, Efficient and Stable Polymer Emitters and their Single Layer Polymer Light Emitting Diodes

Konstantinos C. Andrikopoulos,[1] Despoina Tselekidou,[3] Charalampos Anastasopoulos,[1] Kyparisis Papadopoulos,[3] Vasileios Kyriazopoulos,[4] Stergios Logothetidis,[3,4] Joannis K. Kallitsis,[1,2] Maria Gioti,[3,*] Aikaterini K. Andreopoulou,[1,2,*]

a) Synthesis

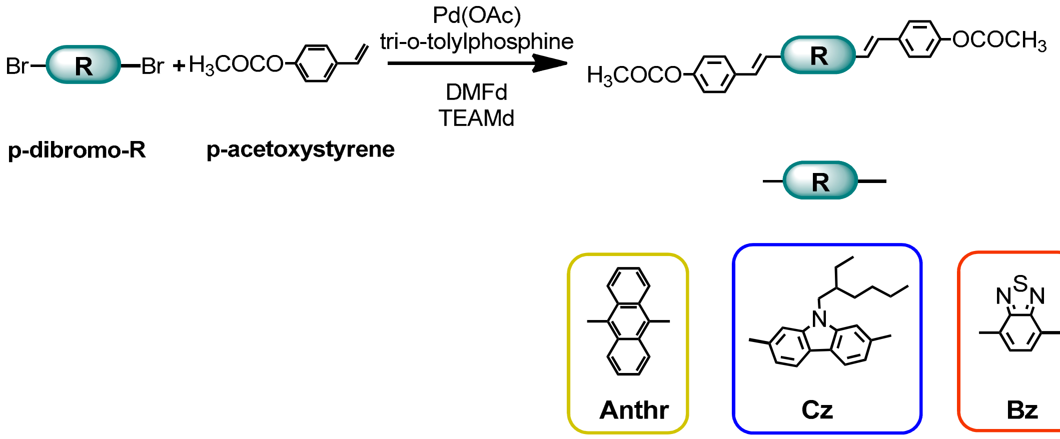

Scheme S1. General synthetic method of fluorescent bis(p-acetoxystyryl) monomers prepared in this study bearing anthracene (**Anthr**), carbazole (**Cz**) and benzothiadiazole (**Bz**) central cores.

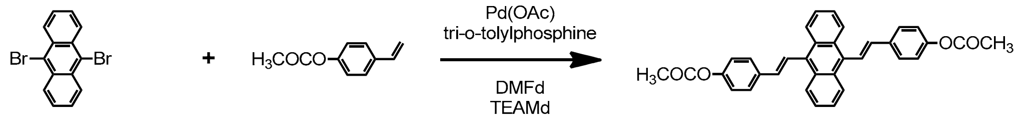

9,11-dibromoanthracene **p-acetoxystyrene** **Anthr**

9,10-bis(p-acetoxystyryl)anthracene (Anthr): To a degassed mixture of 9,10-dibromo-anthracene (50.00 g, 148.71 mmol), p-acetoxy-styrene (68.26 mL, 446.14 mmol), Pd(OAc) (334.00 mg, 1.49 mmol) and tri-o-tolylphosphine (2.72 g, 8.92 mmol) were added, along with triethylamine$_{dry}$ (69.64 mL, 499.67 mmol) and DMF$_{dry}$ (250 mL) and the mixture was heated at 130 °C under argon for 48h under a continuous stream of argon. The reaction mixture was then cooled to r.t. and the formed precipitate was filtered off and washed with methanol. To the filtrate solution, an equal volume of methanol was added, and the filtrate mixture was stored at the refrigerator (6 °C) for 12h forcing more product to precipitate. All solids were combined were recrystallized from toluene. The final product (**Anthr**) was received in 65% yield, 48 g.

^{1}H-NMR ($CDCl_3$, 600 MHz, 298 K) δ (ppm) = 8.37 (dd, 4H), 7.88 (d, 2H), 7.70 (d, 4H), 7.48 (dd, 4H), 7.19 (d, 4H), 6.92 (d, 2H), 2.36 (s, 2H).

^{13}C-NMR ($CDCl_3$, 150 MHz, 298 K) δ (ppm) = 169.51, 150.41, 136.47, 135.11, 132.54, 129.55, 127.55, 126.38, 125.41, 125.32, 122.00, 121.16.

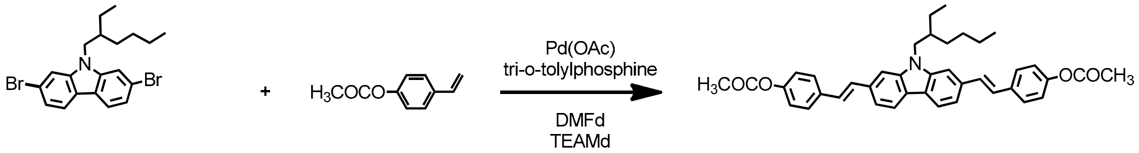

2,7-dibromo-9-(2-ethylhexyl)-9H-carbazole **p-acetoxystyrene** **Cz**

2,7-bis(p-acetoxystyryl)-9-(2-ethylhexyl)-9H-carbazole (Cz): To a degassed mixture of 2,7-dibromo-9-(2-ethylhexyl)-9H-carbazole (20.00 g, 45.74 mmol), p-acetoxy-styrene (18.20 mL, 118.94 mmol), Pd(OAc) (154.00 mg, 0.69 mmol) and tri-o-tolylphosphine (1.25 g, 4.12 mmol) were added, along with triethylamine$_{dry}$ (23.27 mL, 166.97 mmol) and DMF$_{dry}$ (110 mL) and the mixture was heated at 130 °C under argon for 48h under a continuous stream of argon. The mixture was allowed to cool to r.t., 300 mL of

methanol were then added, and the mixture was stirred at r.t. for 5h. The solid thus formed was filtered off, washed with methanol and dried under vacuum at 40 °C for 12h. The crude product was redissolved in chloroform and to that solution activated carbon was added to adsorb any catalyst traces, the mixture was stirred at r.t. for 8h and then filtrated through celite. The filtrate was evaporated under reduced pressure and to the solid that-formed methanol was added. The pale white product formed was filtrated and dried under vacuum at 40 °C. 32 g **(**85% yield) of the pure **Cz** were thus obtained.

^{1}H-NMR ($CDCl_3$, 600 MHz, 298 K) δ (ppm) = 8.02 (d, 2H), 7.57 (d, 4H), 7.43 (d, 4H), 7.26 (d, 2H), 7.19 (d, 2H), 7.12 (d, 4H), 4.19 (m, 2H), 2.32 (s, 6H), 2.13 (t, 1H), 1.42 (m, 6H), 1.32 (m, 2H), 0.97 (t, 3H), 0.91 (t, 3H).

^{13}C-NMR ($CDCl_3$, 150 MHz, 298 K) δ (ppm) = 169.52, 149.49, 141.98, 135.42, 134.94, 130.11, 127.35, 127.00, 122.53, 121.80, 120.39, 117.75, 115.64, 107.22, 47.28, 39.37, 30.95, 28.77, 24.50, 23.06, 21.16, 14.11, 10.99.

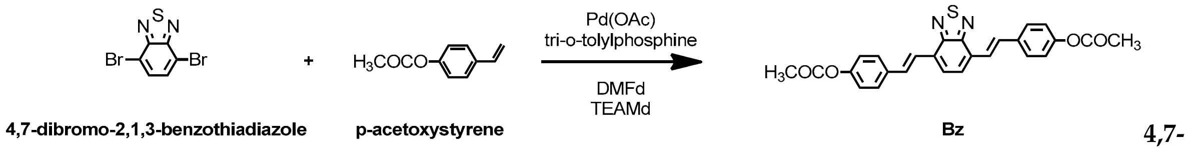

4,7-bis(p-acetoxystyryl)-2,1,3-benzothiadiazole (Bz): To a degassed mixture of 4,7-dibromo-2,1,3-benzothiadiazole (4 g, 13.61 mmol), p-acetoxy-styrene (6.25 mL, 40.82 mmol), Pd(OAc) (30.60 mg, 0.136 mmol) and tri-o-tolylphosphine (249 mg, 0.818 mmol) were added, along with triethylamine(dry) (6.40 mL, 45.70 mmol) and DMF_{dry} (30 mL) and the mixture was heated at 130°C under argon for 48h under a continuous stream of argon. The reaction mixture was then cooled to r.t. and the formed precipitate was filtered off and to the filtrate methanol was added and after cooling to the refrigerator (6 °C) more product precipitated. All solids were combined and washed with methanol. The crude product was recrystallized from toluene. The final pure product (**Bz**) was received in 80% yield, 5 g.

^{1}H-NMR ($CDCl_3$, 600 MHz, 298 K) δ (ppm) = 7.98 (d, 2H), 7.65 (d, 4H), 7.64 (s, 2H), 7.57 (d, 2H), 7.13 (d, 4H), 2.32 (s, 6H).

^{13}C-NMR ($CDCl_3$, 150 MHz, 298 K) δ (ppm) = 169.39, 153.85, 150.48, 135.28, 132.22, 129.24, 127.83, 127.05, 124.70, 121.88, 45.98, 21.15, 8.58

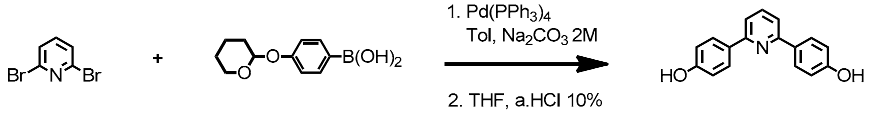

2,6-dibromopyridine **p-acetoxystyrene** **HOpy**

2,6-bis(4-hydroxyphenyl)pyridine (HOpy): To a degassed mixture of 2,6-dibromopyridine (337.71 mmol, 80.00 g), 2-tetrahydropyranyl-oxy-4-phenyl boronic acid (759.89 mmol, 168.70 g), and $Pd(PPh_3)_4$ (4.39 mmol, 5.07 g) were added, along with toluene (1400 mL) and 3 M Na_2CO_3 (2026.25 mmol, 675 mL) under a continuous stream of argon. The mixture was vigorously stirred at reflux for 5h under argon. The organic layer was afterward separated, washed twice with deionized H_2O and the organic solvent was removed under reduced pressure. The residue was redissolved in THF and HCl 37% w/w (150 mL) were then added, and the mixture was stirred for 2h at r.t. The yellow precipitate thus formed was filtered, washed with H_2O until the filtrate reached pH=5, washed again with Na_2CO_3 1.5 M to deprotonate the pyridine groups and finally dried under vacuum at 60 °C. The dried diol product was redissolved in DMF to a concentration of 30% w/w and to that solution activated carbon was added in order to adsorb any catalyst traces. The mixture was filtrated through celite and the filtrate was precipitated in H_2O. The pale white product thus formed was collected via filtration and dried under vacuum at 80 °C. The 2,6-bis(4-hydroxyphenyl)pyridine **(HOpy)** diol was obtained in 85% yield, 75 g.

^{1}H-NMR (DMSO-d_6, 600 MHz, 298 K) δ (ppm) = 9.72 (s, 2H), 8.04 (d, 4H), 7.83 (t, 1H), 7.70 (d, 2H), 6.90 (d, 4H).

^{13}C-NMR (DMSO-d_6, 150 MHz, 298 K) δ (ppm) = 158.97, 155.86, 138.29, 130.24, 128.40, 116.94, 115.92, 31.12.

A general polymerization process is as follows:

For a total of 1 mmol of the monomer derivatives [R1=x mmol, R2=y mmol, and R3=z mmol], (x+y+z) mmol of bis(4-fluorophenyl)sulfone and 2(x+y+z) mmol of K_2CO_3 were placed into a flame dried flask equipped with a magnetic stirrer. DMAc (mL) and toluene (mL) were then added corresponding to a total concentration of 10 %wt for the organic solids and the mixture was stirred under argon until the organic solids were dissolved. The mixture was thoroughly degassed and filled with Ar and finally the flask was fitted with a Dean-Stark apparatus. The polymerization mixture was stirred at 150 °C for 4hrs. Then the temperature was increased to 165 °C and an azeotropic mixture of toluene-water was removed. The reaction was left stirring for another 4 hours, before it was precipitated in a 5fold excess of ethanol. The precipitated polymer was washed with ethanol and was left stirring overnight in ethyl acetate. The polymer was then filtered, dried under vacuum at 50 °C for 12hr and then stirred in warm water for 24hr. The polymer was collected via filtration, washed thoroughly with warm water and ethanol and was then dried under vacuum at 50 °C. The polymer was redissolved in NMP, and the solution was passed through a short column filled with silica gel to remove any inorganic remainings. The final purified polymer solution was casted in a Petri dish at 80 °C until complete evaporation of the solvent and finally dried at 80°C under vacuum for at least 12hr. The reaction yield was 85-95% in all cases.

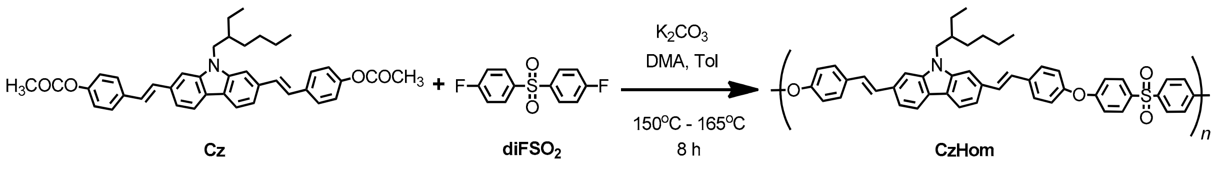

CzHom: To a flame dried flask equipped with a magnetic stirrer were added 2,7-bis(p-acetoxystyryl)-9-(2-ethylhexyl)-9H-carbazole (0.005 mol, 3.000 g), bis(4-fluorophenyl)sulfone (0.005 mol, 1.270 g), K_2CO_3 (0.0075 mol, 1.04 g), DMAc (12 mL) and toluene (1 mL) and the mixture was stirred under argon until the organic solids were dissolved. The mixture was thoroughly degassed and filled with Ar and finally the

flask was fitted with a Dean-Stark apparatus. The polymerization mixture was stirred at 150 °C for 4hrs. Then the temperature was increased to 165 °C and an azeotropic mixture of toluene-water was removed. The reaction was left stirring for another 4 hours, before it was precipitated in a 5fold excess of ethanol. The precipitated polymer was treated as described above for the general polymerization procedure.

^{1}H-NMR (DMF-d$_7$, 600 MHz, 298 K) δ (ppm) = 8.19 (b, 2H), 8.11 (d, 2H), 8.07 (d, 4H), 7.80 (d, 4H), 7.58 (b, 2H), 7.53 (d, 2H), 7.26 (b, 4H), 7.23 (b, 6H), 4.43 (b, 2H), 2.20 (b, 1H), 1.50-1.17 (b, 8H), 0.95 (b, 3H), 0.83 (b, 3H).

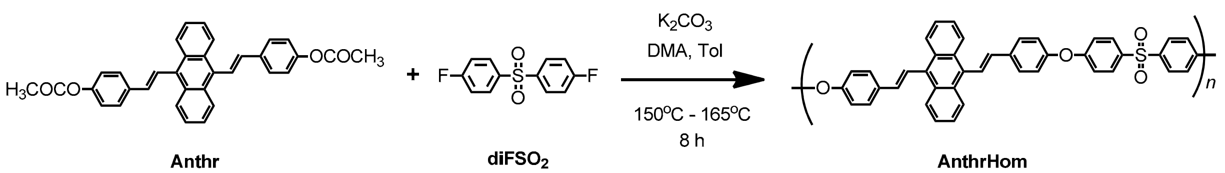

AnthrHom: To a flame dried flask equipped with a magnetic stirrer were added 9,10-bis(p-acetoxystyryl)anthracene (**Anthr**) (0.0802 mmol, 400 mg), bis(4-fluorophenyl)sulfone (0.0802 mmol, 204 mg), K_2CO_3 (0.1610 mmol, 222 mg), DMAc (8 mL) and toluene (2 mL) and the mixture was stirred under argon until the organic solids were dissolved. The mixture was thoroughly degassed and filled with Ar and finally the flask was fitted with a Dean-Stark apparatus. The polymerization mixture was stirred at 150 °C for 4hrs. Then the temperature was increased to 165 °C and an azeotropic mixture of toluene-water was removed. The reaction was left stirring for another 4 hours, before it was precipitated in a 5fold excess of ethanol. The precipitated polymer was treated as described above for the general polymerization procedure.

^{1}H-NMR ($CDCl_3$, 600 MHz, 298 K) δ (ppm) = 8.38 (b, 4H), 8.00- 7.83 (b, 6H), 7.74- 7.65 (b, 4H), 7.54- 7.38 (b, 4H), 7.17- 7.02 (b, 8H), 6.98- 6.79 (b, 2H).

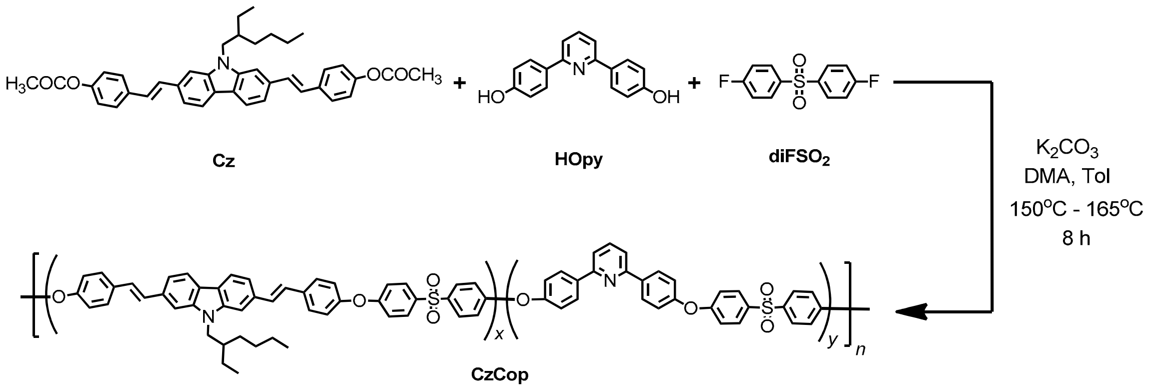

CzCop x/y: To a flame dried flask equipped with a magnetic stirrer were added (x) mmol 2,7-bis(p-acetoxystyryl)-9-(2-ethylhexyl)-9H-carbazole (**Cz**), (y) mmol 2,6-bis(4-hydroxyphenyl)pyridine (**HOpy**), (x+y) mmol bis(4-fluorophenyl)sulfone (**diFSO$_2$**), [2*(x+y)] mmol K_2CO_3, DMAc and toluene and the mixture was stirred under argon until the organic solids were dissolved. The mixture was thoroughly degassed and filled with Ar and finally the flask was fitted with a Dean-Stark apparatus. The polymerization mixture was stirred at 150 °C for 4hrs. Then the temperature was increased to 165 °C and an azeotropic mixture of toluene-water was removed. The reaction was left stirring for another 4 hours, before it was precipitated in a 5fold excess of ethanol. The precipitated polymer was treated as described above for the general polymerization procedure.

CzCop 70/30: ^{1}H-NMR ($CDCl_3$, 600 MHz, 298 K) δ (ppm) = 8.19 (b, 4H), 8.05 (b, 2H), 7.96 (d, 8H), 7.92 (d, 8H), 7.87 (b, 1H), 7.70 (b, 2H), 7.61 (b, 4H), 7.47 (b, 4H), 7.31- 7.25 (b, 4H), 7.25- 7.21 (b, 4H), 7.21- 7.15 (b, 4H), 7.15- 7.04 (b, 4H), 4.22 (b, 2H), 2.15 (b, 1H), 1.53- 1.24 (b, 8H), 0.98 (b, 3H), 0.92(b, 3H).

CzCop 50/50: ^{1}H-NMR ($CDCl_3$, 600 MHz, 298 K) δ (ppm) = 8.15 (b, 4H), 8.02 (s, 2H), 7.92- 7.83 (b, 11H), 7.68 (b, 2H), 7.58 (b, 4H), 7.44 (b, 4H), 7.29- 7.22 (b, 2H), 7.22- 7.18 (b, 4H), 7.18- 7.11 (b, 4H), 7.11- 6.98 (b, 4H), 4.18 (b, 2H), 2.11 (b, 1H), 1.51- 1.00 (b, 8H), 1.00- 0.64 (b, 6H).

CzCop 30/70: ^{1}H-NMR ($CDCl_3$, 600 MHz, 298 K) δ (ppm) = 8.17 (b, 4H), 8.01 (b, 2H), 7.89 (b, 10H), 7.81 (b, 1H), 7.66 (t, 2H), 7.57 (b, 4H), 7.43 (b, 4H), 7.29- 7.22 (b, 2H), 7.22- 7.18 (b, 4H), 7.18- 7.11 (b, 4H), 7.11- 7.01 (b, 4H), 4.19 (b, 2H), 2.11 (b, 1H), 1.49- 1.23 (b, 8H), 0.94 (b, 3H), 0.88 (b, 3H).

CzCop 10/90: ^{1}H-NMR ($CDCl_3$, 600 MHz, 298 K) δ (ppm) = = 8.22- 8.11 (b, 4H), 8.01 (b, 2H), 7.93- 7.84 (b, 10H), 7.84- 7.78 (b, 1H), 7.73- 7.62 (b, 2H), 7.58 (b, 4H), 7.46- 7.38 (b, 4H), 7.22- 7.11 (b, 8H), 7.11- 6.99 (b, 10H), 4.20 (b, 2H), 2.13 (b, 1H), 1.47- 1.23 (b, 8H), 0.98- 0.77 (b, 6H).

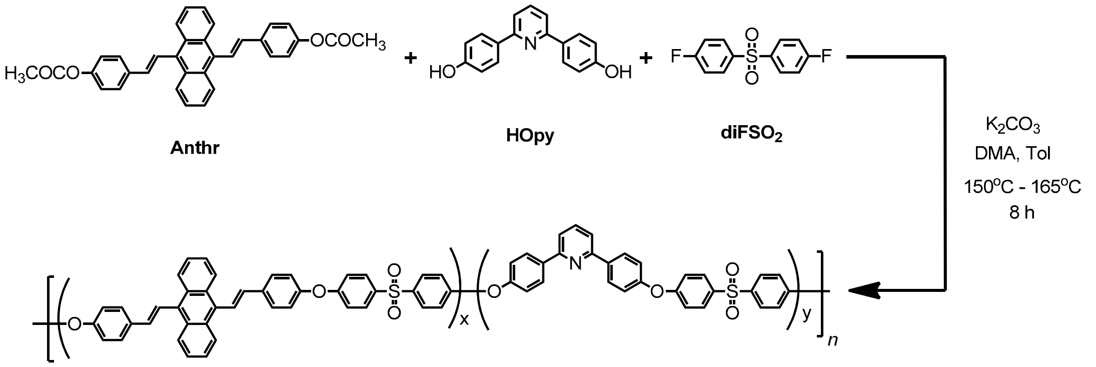

AnthrCop

AnthrCop: To a flame dried flask equipped with a magnetic stirrer were added (x) mmol 9,10-bis(p-acetoxystyryl)anthracene (**Anthr**), (y) mmol 2,6-Bis(4-hydroxyphenyl)pyridine (**HOpy**), (x+y) mmol bis(4-fluorophenyl)sulfone (**diFSO$_2$**), [2*(x+y)] mmol K_2CO_3, DMAc and toluene and the mixture was stirred under argon until the organic solids were dissolved. The mixture was thoroughly degassed and filled with Ar and finally the flask was fitted with a Dean-Stark apparatus. The polymerization mixture was stirred at 150 °C for 4hrs. Then the temperature was increased to 165 °C and an azeotropic mixture of toluene-water was removed. The reaction was left stirring for another 4 hours, before it was precipitated in a 5fold excess of ethanol. The precipitated polymer was treated as described above for the general polymerization procedure.

AnthrCop 70/30 ^{1}H-NMR ($CDCl_3$, 600 MHz, 298 K) δ (ppm) = 8.37 (b, 4H), 8.16 (b, 4H), 7.99- 7.78 (b, 10H), 7.73- 7.56 (b, 6H), 7.56- 7.36 (b, 4H), 7.22- 6.98 (b, 16H), 6.98- 6.78 (b, 2H).

AnthrCop 50/50 ^{1}H-NMR ($CDCl_3$, 600 MHz, 298 K) δ (ppm) = 8.37 (b, 4H), 8.15 (b, 4H), 7.99- 7.78 (b, 11H), 7.74- 7.60 (b, 6H), 7.54- 7.37 (b, 4H), 7.22- 6.98 (b, 16H), 6.98- 6.84 (b, 2H).

AnthrCop 30/70 ^{1}H-NMR ($CDCl_3$, 600 MHz, 298 K) δ (ppm) = 8.37 (b, 4H), 8.18- 8.12 (b, 4H), 7.97- 7.84 (b, 10H), 7.81 (b, 1H), 7.73- 7.58 (b, 6H), 7.58- 7.38 (b, 4H), 7.22- 6.96 (b, 16H) 6.96- 6.76 (b, 2H).

AnthrCop 10/90 ^{1}H-NMR ($CDCl_3$, 600 MHz, 298 K) δ (ppm) = 8.33 (b, 4H), 8.23- 8.12 (b, 4H), 7.98- 7.82 (b, 10H), 7.82- 7.78 (b, 1H), 7.74- 7.64 (b, 6H), 7.21- 7.01 (b, 16H), 6.97- 6.76 (b, 2H).

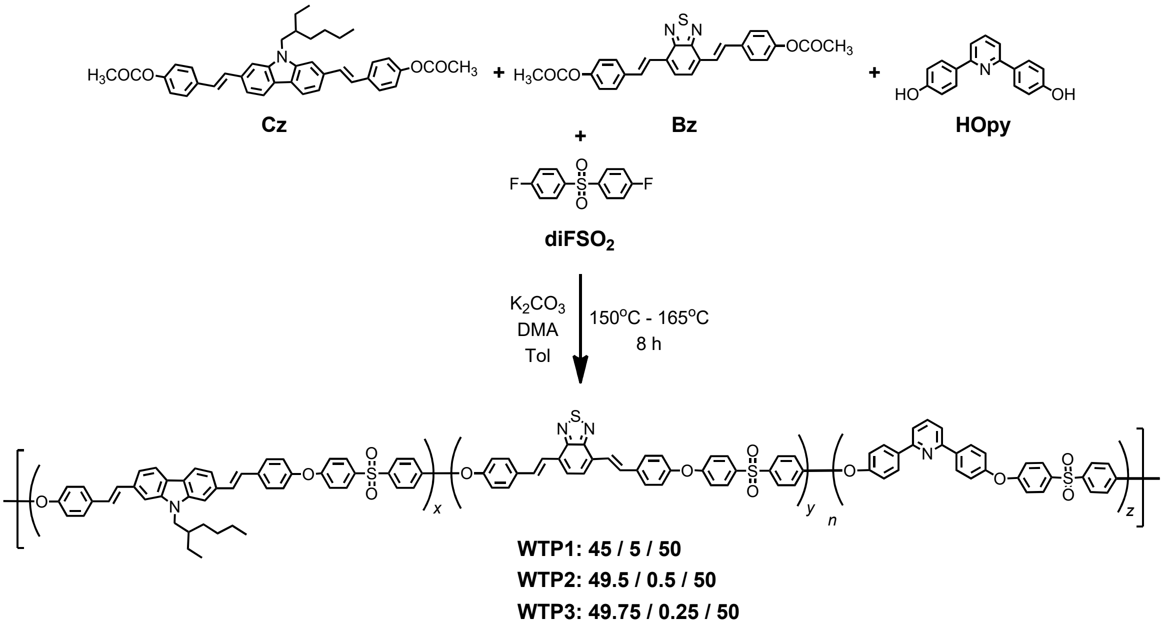

WTP1 x=45, y=5, z=50: To a flame dried flask equipped with a magnetic stirrer were added 2,7-bis(p-acetoxystyryl)-9-(2-ethylhexyl)-9H-carbazole (**Cz**) (0.099 mmol, 59.120 mg), 4,7-bis(p-acetoxystyryl)-2,1,3-benzothiadiazole (**Bz**) (0.011 mmol, 5 mg), 2,6-bis(4-hydroxyphenyl)pyridine (**HOpy**) (0.110 mmol, 28.850 mg), bis(4-fluorophenyl)sulfone (**diFSO$_2$**) (0.220 mmol, 35.740 mg), K_2CO_3 (0.274 mmol, 37.870 mg), DMAc (1.5 mL) and toluene (0.5 mL) and the mixture was stirred under argon until the organic solids were dissolved. The mixture was thoroughly degassed and filled with Ar and finally the flask was fitted with a Dean-Stark apparatus. The polymerization mixture was stirred at 150 °C for 4hrs. Then the temperature was increased to 165 °C and an azeotropic mixture of toluene-water was removed. The reaction was left stirring for another 4 hours, before it was precipitated in a 5fold excess of ethanol. The precipitated polymer was treated as described above for the general polymerization procedure.

^{1}H-NMR ($CDCl_3$, 600 MHz, 298 K) δ (ppm) = 8.17 (b, 4H), 8.03 (b, 2H), 7.98- 7.86 (b, 16H), 7.86- 7.80 (b, 1H), 7.71- 7.64 (b, 2H), 7.63- 7.54 (b, 10H), 7.49- 7.39 (b, 4H), 7.30- 7.23 (b, 4H), 7.23- 7.13 (b, 12H), 7.13- 7.01 (b, 10H), 4.29- 4.12 (b, 2H), 2.17- 2.08 (b, 1H), 1.52- 1.23 (b, 8H), 1.01- 0.82 (b, 6H).

WTP2 x=49.5, y=0.5, z=50: To a flame dried flask equipped with a magnetic stirrer were added 2,7-bis(p-acetoxystyryl)-9-(2-ethylhexyl)-9H-carbazole (**Cz**) (0.003 mol, 1.500 g), 4,7-bis(p-acetoxystyryl)-2,1,3-benzothiadiazole (**Bz**) (0.253 mmol, 11.500 mg), 2,6-bis(4-hydroxyphenyl)pyridine (**HOpy**) (2.526 mmol, 664.15 mg), bis(4-fluorophenyl)sulfone (**diFSO$_2$**) (5.053 mmol, 1.280 g), K_2CO_3 (1.112 mmol, 1.540 g), DMAc (35 mL) and toluene (8 mL) and the mixture was stirred under argon until the organic solids were dissolved. The mixture was thoroughly degassed and filled with Ar and finally the flask was fitted with a Dean-Stark apparatus. The polymerization mixture was stirred at 150 °C for 4 hours. Then the temperature was increased to 165 °C and an azeotropic mixture of toluene-water was removed. The reaction was left stirring for another 4 hours, before it was precipitated in a 5fold excess of ethanol. The precipitated polymer was treated as described above for the general polymerization procedure.

^{1}H-NMR ($CDCl_3$, 600 MHz, 298 K) δ (ppm) = 8.18 (b, 4H), 8.04 (b, 2H), 7.97- 7.83 (b, 17H), 7.73- 7.65 (b, 2H), 7.65- 7.55 (b, 10H), 7.50- 7.41 (b, 4H), 7.31- 7.25 (b, 4H), 7.24- 7.14 (b, 12H), 7.14- 7.01 (b, 10H), 4.21 (b, 2H), 2.18- 2.08 (b, 1H), 1.53- 1.22 (b, 8H), 1.02- 0.94 (b, 3H), 0.94- 0.80 (b, 3H).

WTP3 x=49.75, y=0.25, z=50: To a flame dried flask equipped with a magnetic stirrer were added 2,7-bis(p-acetoxystyryl)-9-(2-ethylhexyl)-9H-carbazole (**Cz**) (1.667 mmol, 1.000 g), 4,7-bis(p-acetoxystyryl)-2,1,3-benzothiadiazole (**Bz**) (0.008 mmol, 3.820 mg), 2,6-bis(4-hydroxyphenyl)pyridine (**HOpy**) (1.676 mmol, 441.200 mg), bis(4-fluorophenyl)sulfone (**diFSO$_2$**) (3.351 mmol, 852.10 mg), K_2CO_3 (7.373 mmol, 1.019 g), DMAc (25 mL) and toluene (5 mL) and the mixture was stirred under argon until the organic solids were dissolved. The mixture was thoroughly degassed and filled with Ar and finally the flask was fitted with a Dean-Stark apparatus. The polymerization mixture was stirred at 150 °C for 4hrs. Then the temperature was increased to 165 °C and an azeotropic mixture of toluene-water was removed. The reaction

was left stirring for another 4 hours, before it was precipitated in a 5fold excess of ethanol. The precipitated polymer was treated as described above for the general polymerization procedure.

^{1}H-NMR ($CDCl_3$, 600 MHz, 298 K) δ (ppm) = 8.20- 8.12 (b, 4H), 8.05- 7.98 (b, 2H), 7.93- 7.87 (b, 16H), 7.87- 7.81 (b, 1H), 7.71-7.64 (b, 2H), 7.62- 7.54 (b, 10H), 7.48- 7.40 (b, 4H), 7.29- 7.23 (b, 4H), 7.23- 7.12 (b, 12H), 7.12- 7.00 (b, 10H), 4.30- 4.10 (b, 2H), 2.17- 2.07 (b, 1H), 1.52- 1.19 (b, 8H), 0.99- 0.92 (b, 3H), 0.92- 0.86 (b, 3H).

b) NMR spectra

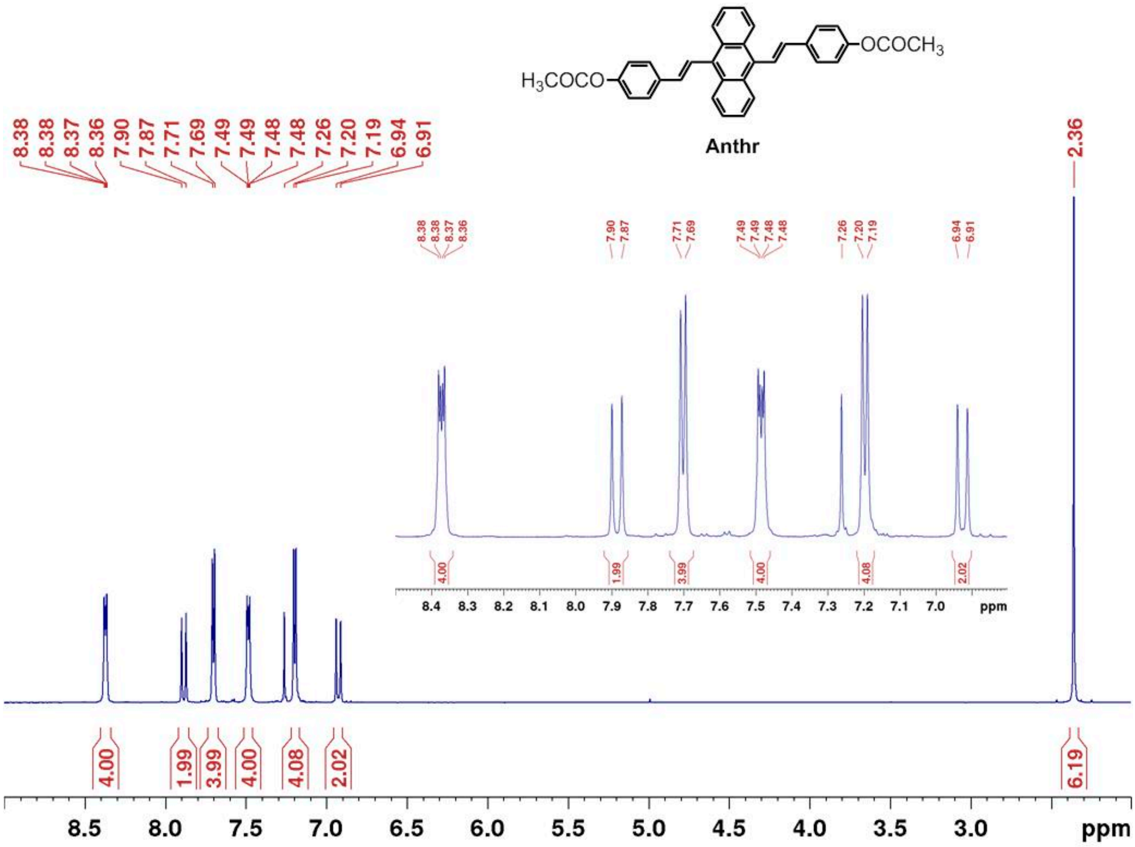

Figure S1: ^{1}H-NMR spectrum of **9,10-bis(p-acetoxystyryl)anthracene (Anthr)** in $CDCl_3$. The inset shows the aromatic protons area in magnification.

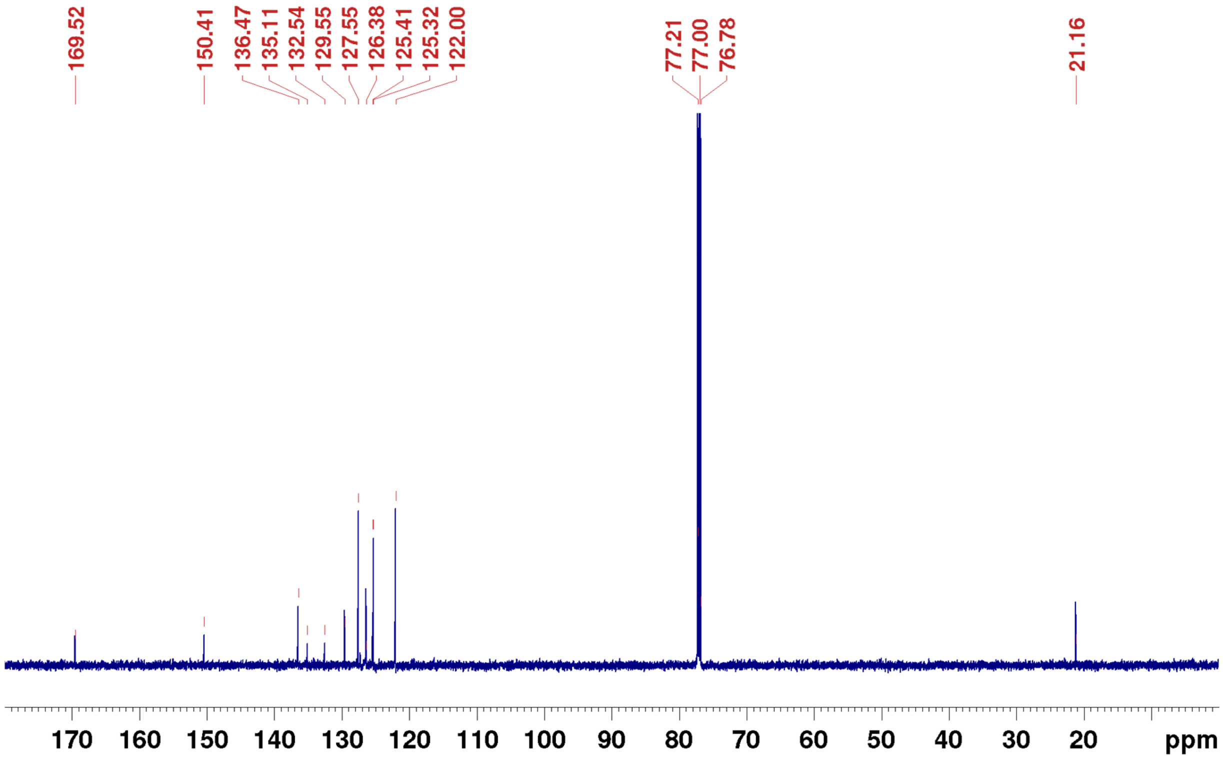

Figure S2: ^{13}C-NMR spectrum of **9,10-bis(p-acetoxystyryl)anthracene (Anthr)** in $CDCl_3$.

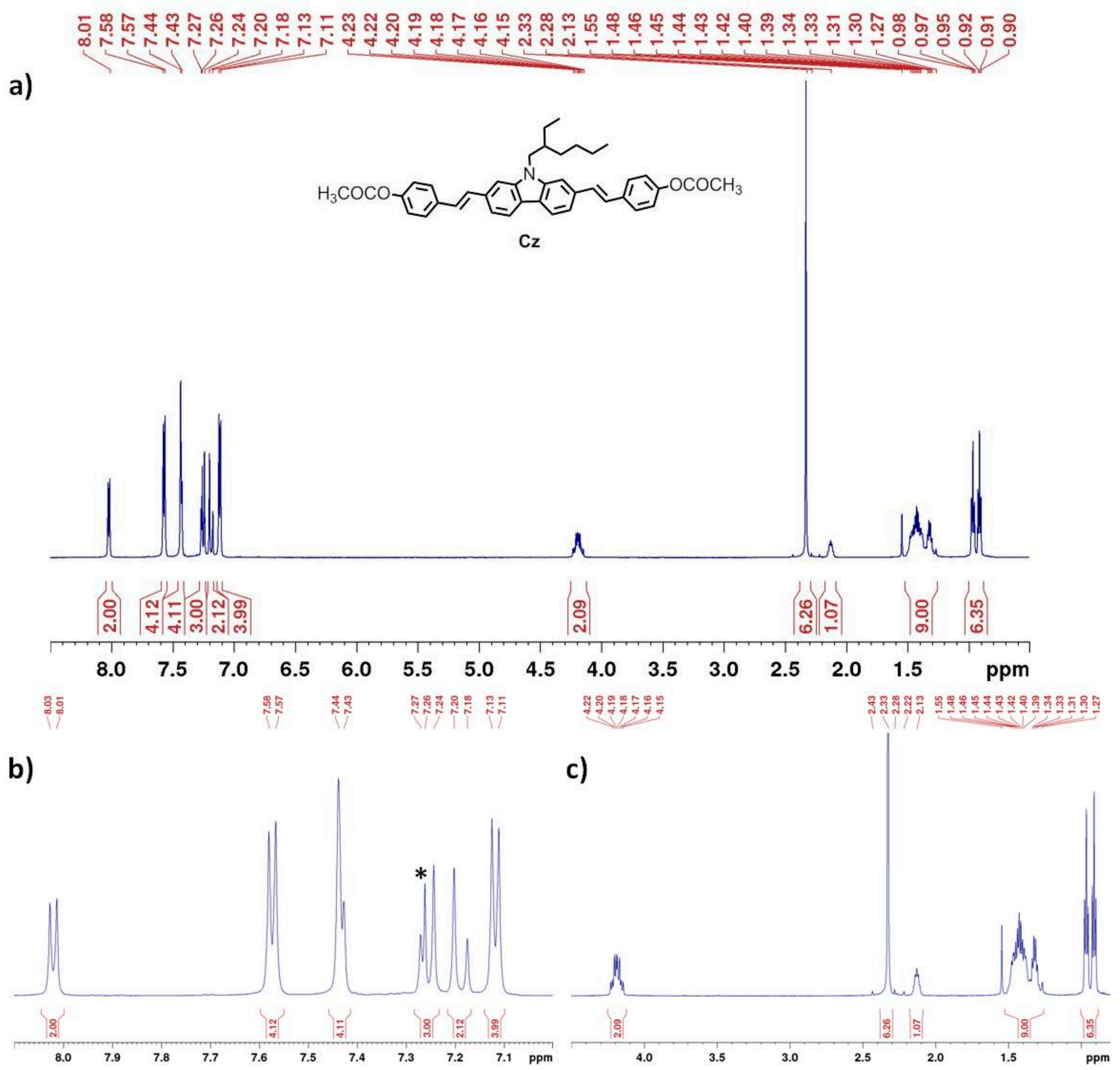

Figure S3: ¹H-NMR spectrum of (a) **2,7-bis(p-acetoxystyryl)-9-(2-ethylhexyl)-9H-carbazole (Cz)** in $CDCl_3$, b) and c) show the aromatic and aliphatic protons areas, respectively, in magnification.

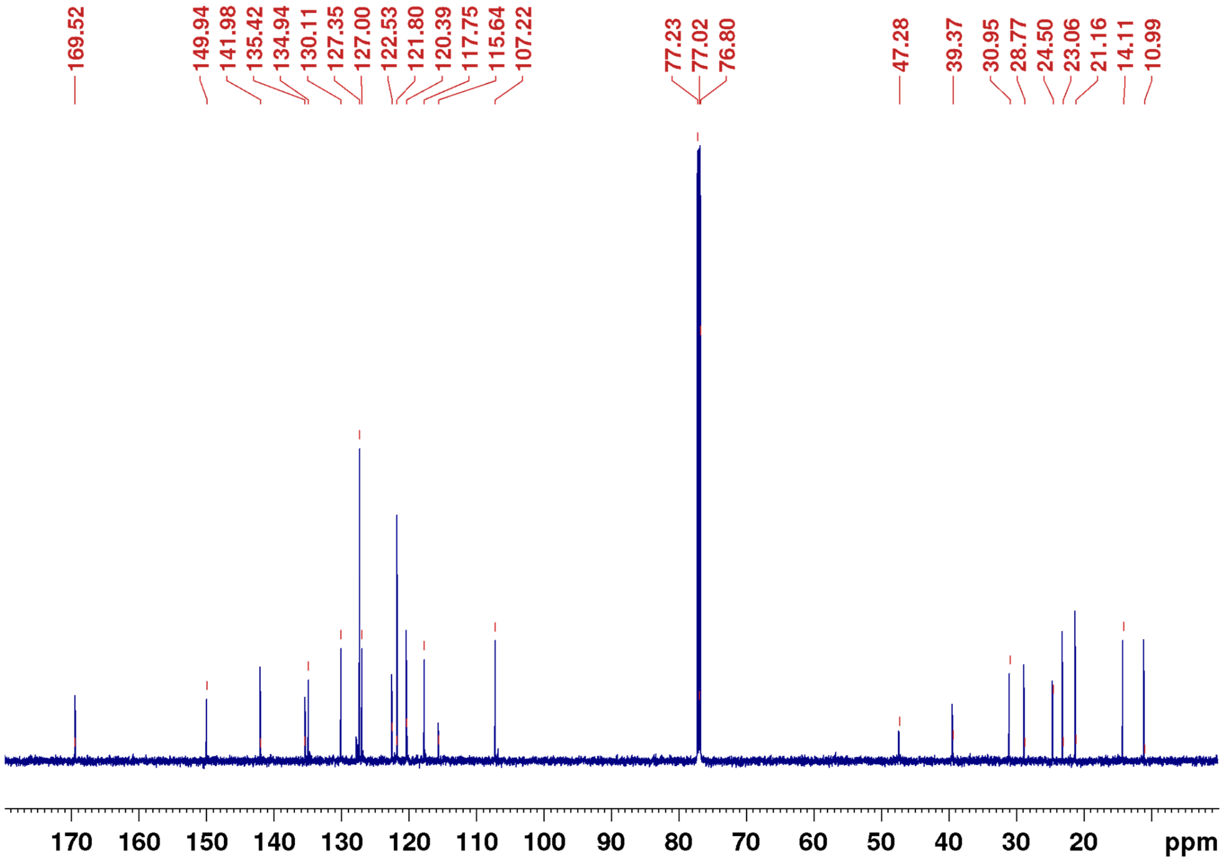

Figure S4: ^{13}C-NMR spectrum of **2,7-bis(p-acetoxystyryl)-9-(2-ethylhexyl)-9H-carbazole (Cz)** in $CDCl_3$,

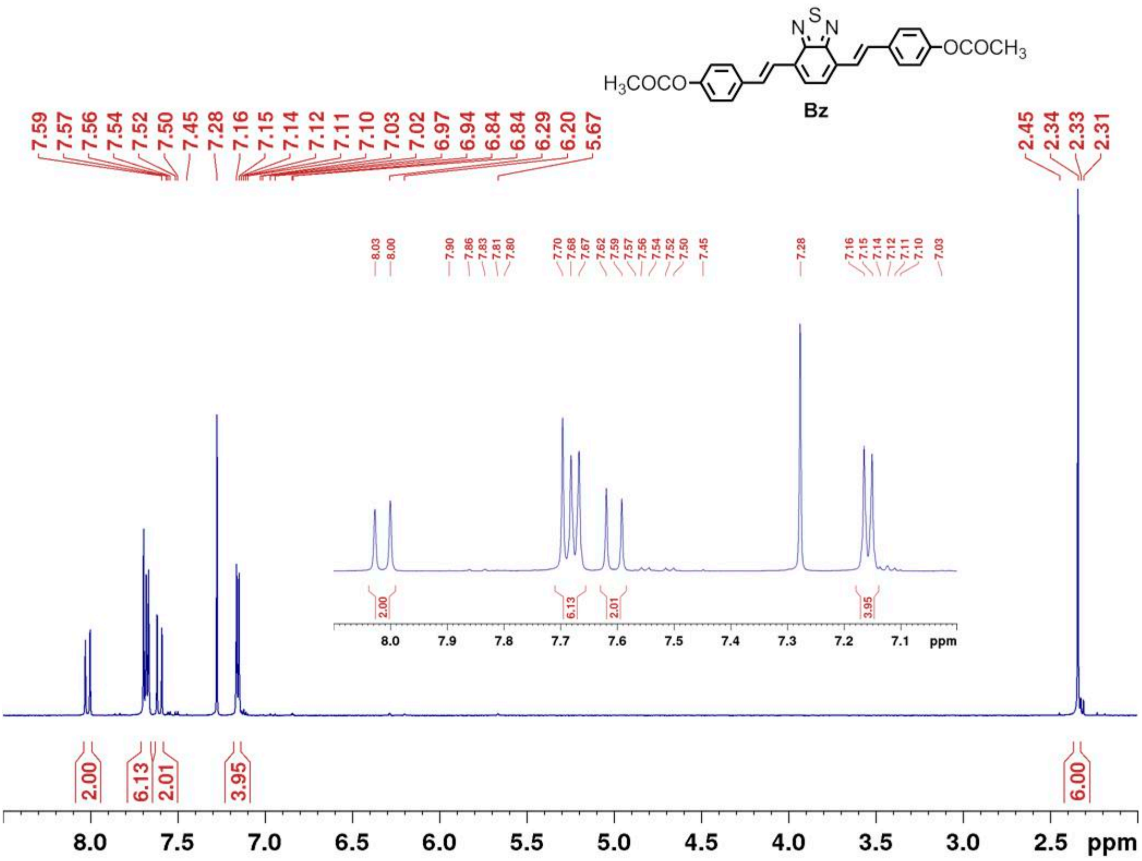

Figure S5: ^{1}H-NMR spectrum of **4,7-bis(p-acetoxystyryl)-2,1,3-benzothiadiazole (Bz)** in $CDCl_3$. The inset shows the aromatic protons area in magnification.

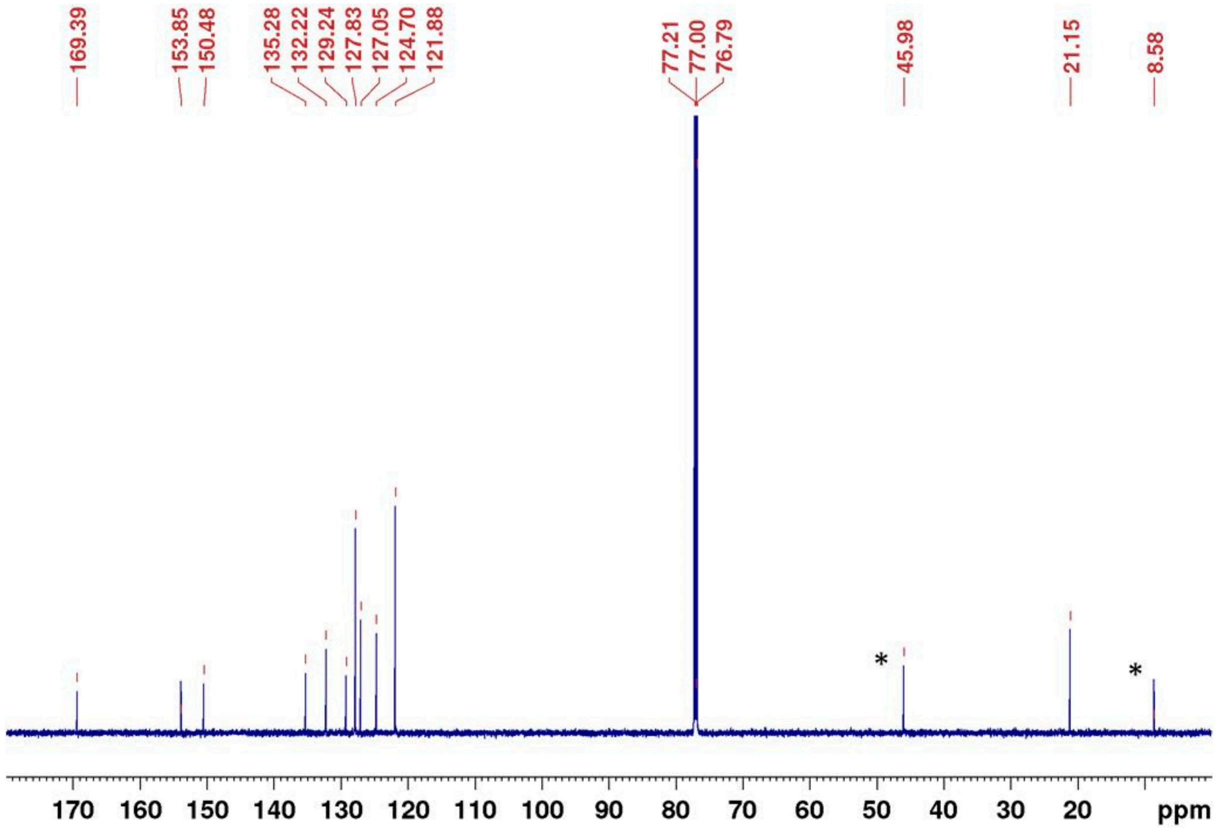

Figure S6: ^{13}C-NMR spectrum of **4,7-bis(p-acetoxystyryl)-2,1,3-benzothiadiazole (Bz)** in $CDCl_3$. The asterisks denote residual solvent impurities.

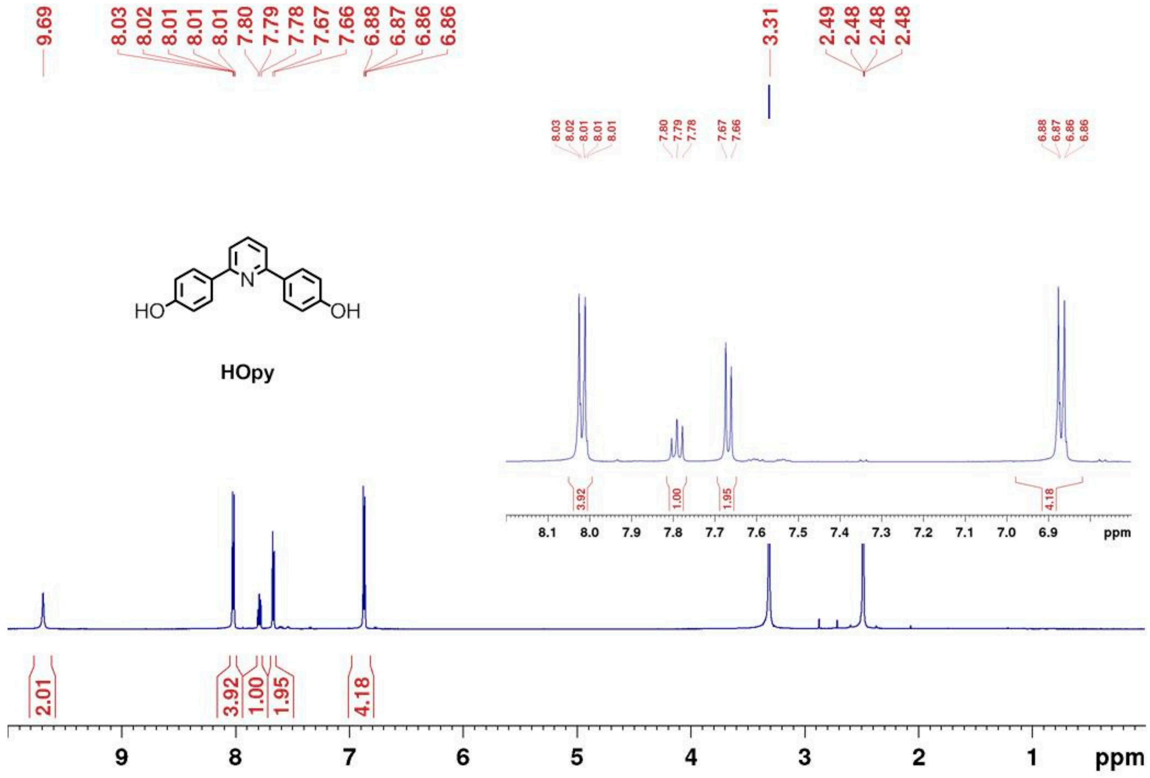

Figure S7: ^{1}H-NMR spectrum of **2,6-bis(4-hydroxyphenyl)pyridine (HOpy)** in DMSO-d6. The inset shows the aromatic protons area in magnification.

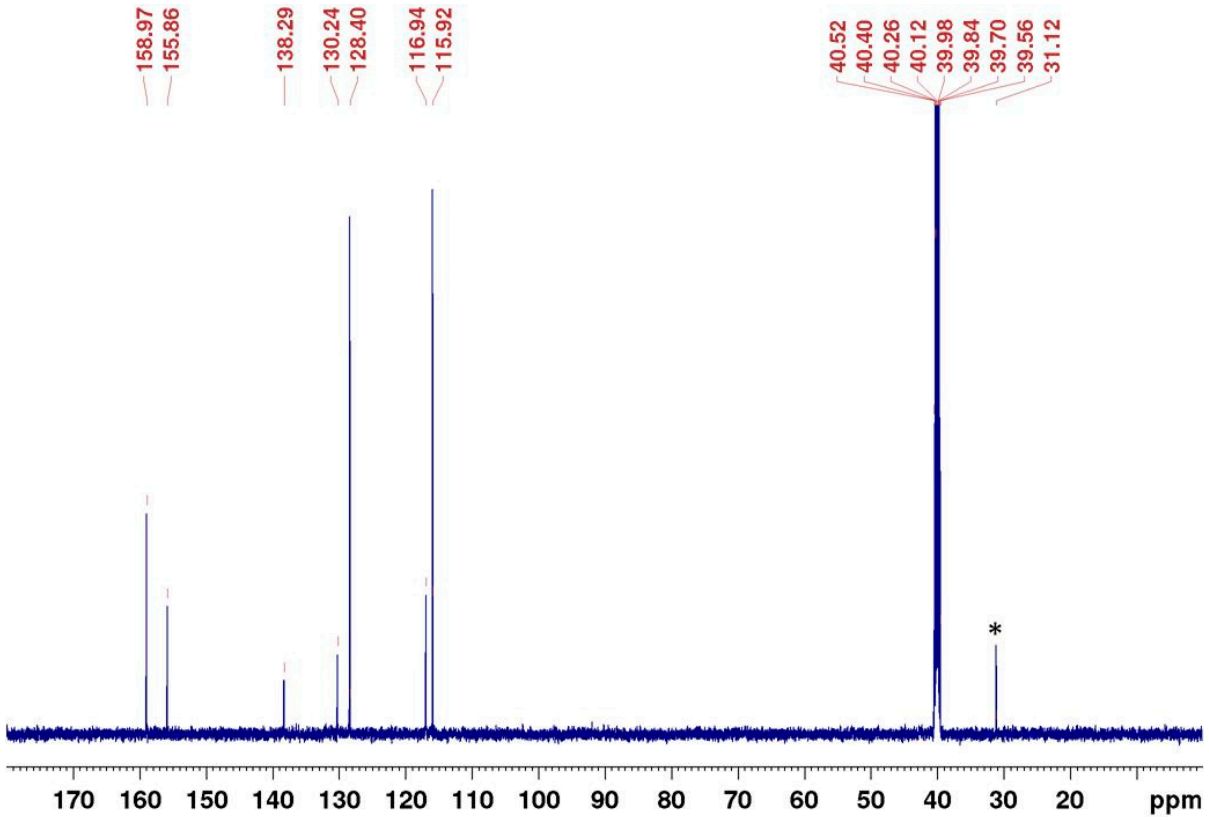

Figure S8: ^{13}C-NMR spectrum of **2,6-bis(4-hydroxyphenyl)pyridine (HOpy)** in DMSO-d_6. The asterisk denotes residual solvent impurity.

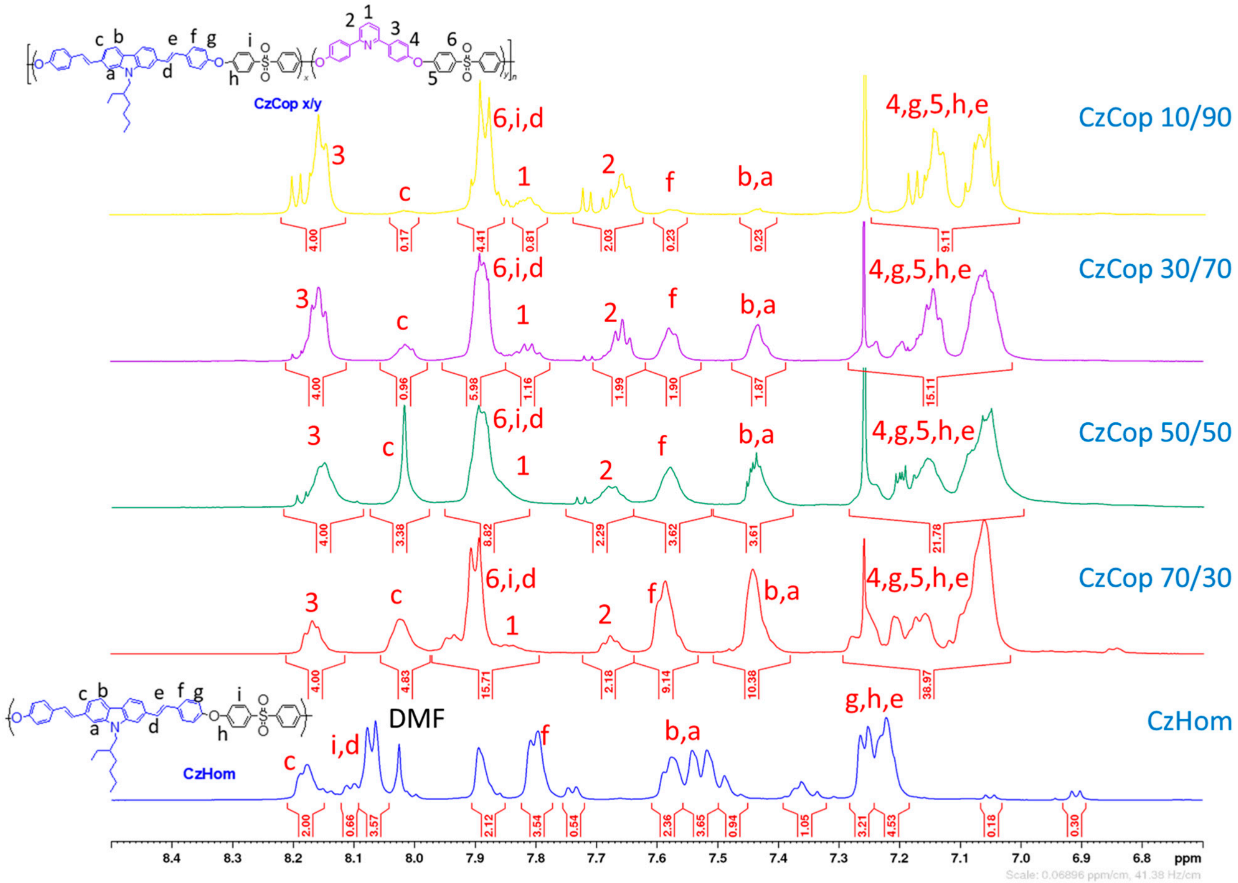

Figure S9: ^{1}H-NMR spectra of the **Cz** based copolymers and of the **CzHom**. All spectra were recorded in $CDCl_3$, except the **CzHom** spectrum for which DMF-d_7 was used.

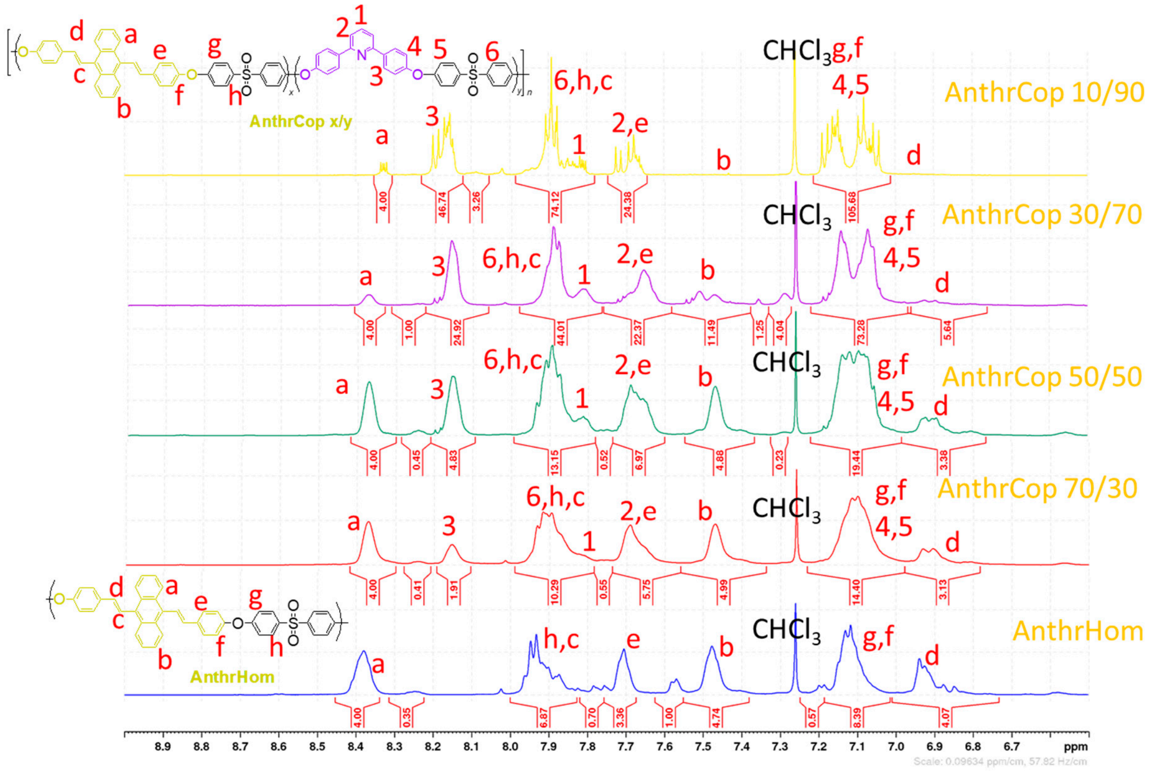

Figure S10: ^{1}H-NMR spectra of the **Anthr** based copolymers and of the **AnthrHom.** All spectra were recorded in $CDCl_3$.

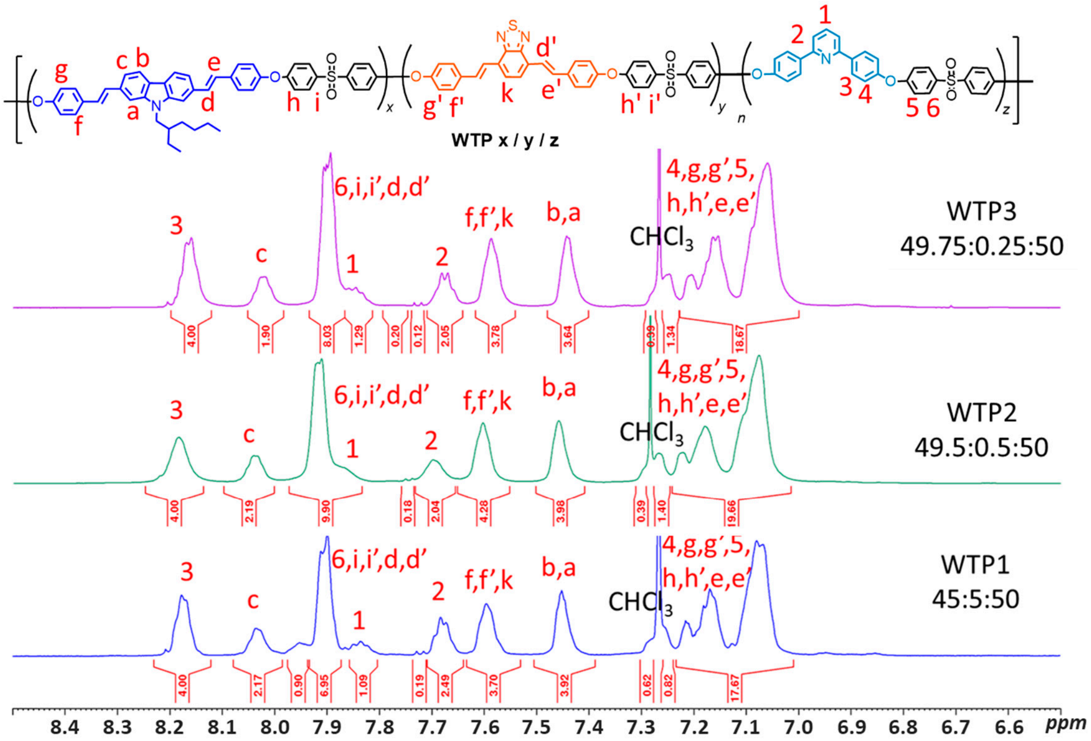

Figure S11. ^{1}H NMR spectra of the **WTPs** white light terpolymers, in $CDCl_3$.

c) Detailed Molecular weight Characteristics

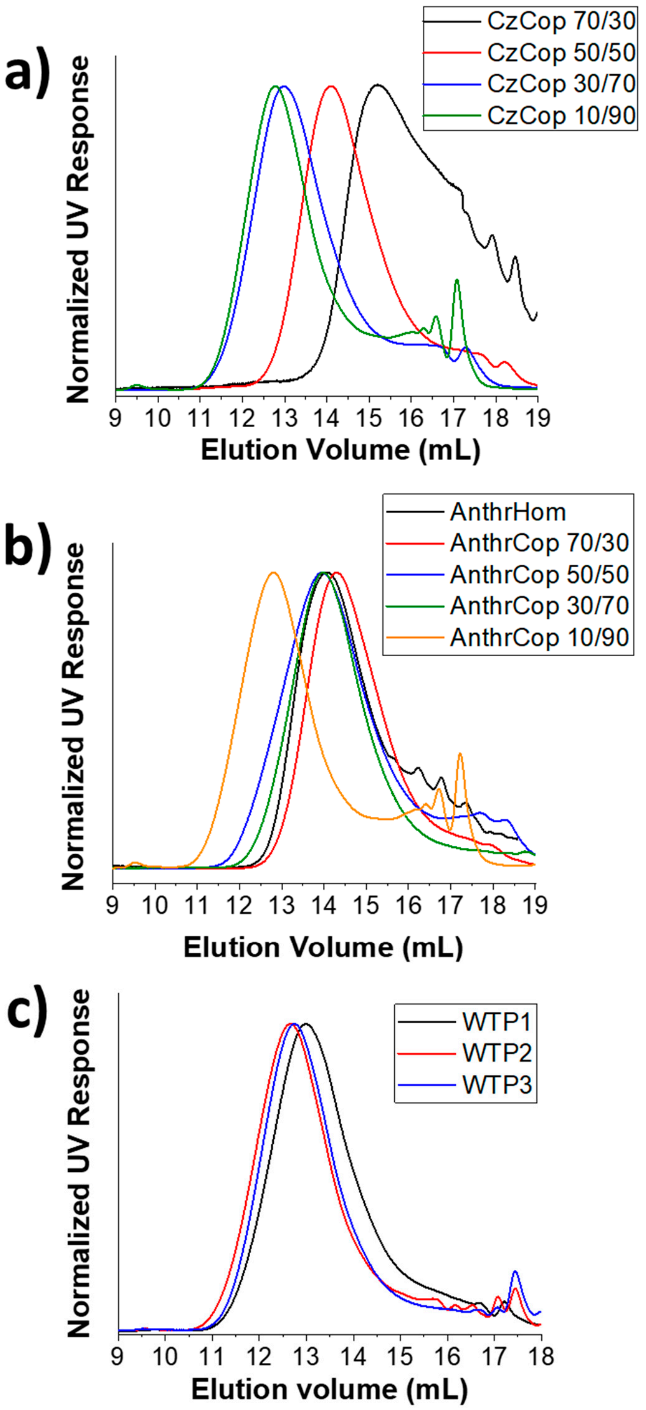

Figure S12: Normalized GPC curves of the **Cz, Anthr** copolymers **(a, b** respectively**)** and of the white light terpolymers (**WTP**) (**c**) luminescent polymers of **Tables 1** and **2.**

Table S1. Percentage feed ratios of the **Cz** and of the **HOpy** monomers in the luminescent **CzHom** and **CzCop** polymers and molecular weight characteristics via GPC.

Code	Chromophore (%)	HOpy (%)	Mp[a], Mn[b] (kDa)	Disp[c]
**CzHom[d]**	100	0	3.00, 2.00	1.17
	100	0	3.50, 3.00	1.15
**CzCop 70/30**	70	30	11.90, 6.90	1.80
**CzCop 50/50**	50	50	6.80, 5.60	1.90
	50	50	11.50, 6.30	1.83
	50	50	19.80, 11.35	2.08
	50	50	23.60, 14.30	1.80
	50	50	51.20, 31.70	1.70
**CzCop 30/70**	30	70	50.50, 34.70	1.77
	30	70	66.60, 23.80	2.80
**CzCop 10/90**	10	90	87.70, 55.80	1.82

a) Mp=peak molecular weight at the peak of the distribution curve

b) Mn=number-average molecular weight

c) Disp=polydispersity Mw/Mn

d) Only the soluble in $CHCl_3$ fractions could be measured

Table S2. Percentage feed ratios of the **Anthr** and of the **HOpy** monomers in the luminescent **AnthrHom** and **AnthrCop** polymers and molecular weight characteristics via GPC.

Code	Chromophore (%)	HOpy (%)	Mp[a], Mn[b] (kDa)	Disp[c]
**AnthrHom**	100	0	2.40, 1.50	1.17
			1.90, 1.60	1.10
**AnthrCop 70/30**	70	30	12.90, 7.30	1.82
			28.80, 11.50	2.90
**AnthrCop 50/50**	50	50	9.10, 4.40	2.32
			15.00, 9.80	1.68
			30.40, 25.00	1.40
			39.00, 19.90	2.00
			55.00, 26.30	2.10
			66.80, 47.00	1.60
**AnthrCop 30/70**	30	70	89.80, 56.80	1.90
**AnthrCop 10/90**	10	90	30.20, 18.50	2.1

a) Mp=peak molecular weight at the peak of the distribution curve

b) Mn=number-average molecular weight

c) Disp=polydispersity Mw/Mn

Table S3. Percentage feed ratios of the **Cz** and **Bz** fluorescent monomers and of the **HOpy** monomer in the white light terpolymers (**WTP**) and molecular weight characteristics via GPC.

Code	Cz (%)	Bz (%)	HOpy (%)	Mp[a], Mn[b] (kDa)	Disp[c]
**WTP1**	45	5	50	11.00, 7.30	1.60
**WTP2**	49.5	0.5	50	20.70, 14.00	1.64
			50	29.20, 20.30	1.60
			50	54.60, 38.20	1.47
			50	66.20, 45.10	1.55
			50	88.80, 68.10	1.63
**WTP3**	49.75	0.25	50	6.60, 3.40	1.97
			50	29.30, 24.40	2.00

a) Mp=peak molecular weight at the peak of the distribution curve

b) Mn=number-average molecular weight

c) Disp=polydispersity Mw/Mn

d) Thermogravimetric Analyses

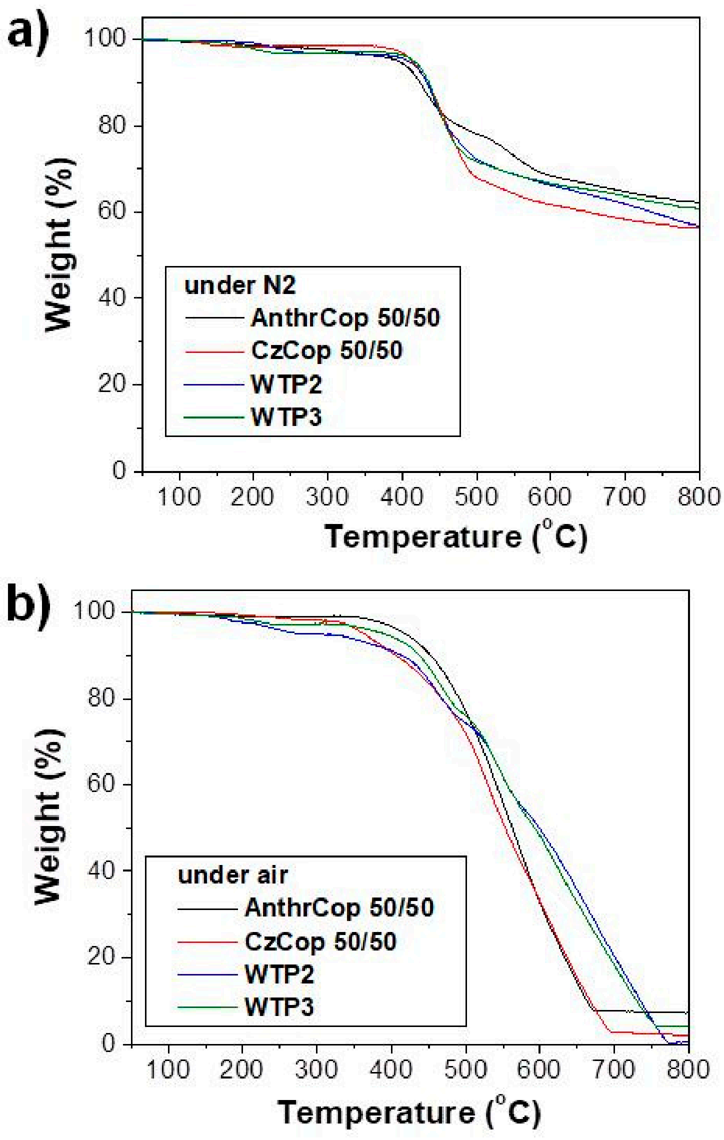

Figure S13. Thermogravimetric analyses curves of **AnthrCop 50/50**, **CzCop 50/50**, **WTP4** and **WTP5** under N_2 (**a**) and under air (**b**), at 10 °C/min.

e) Optoelectronic and Electrochemical Properties

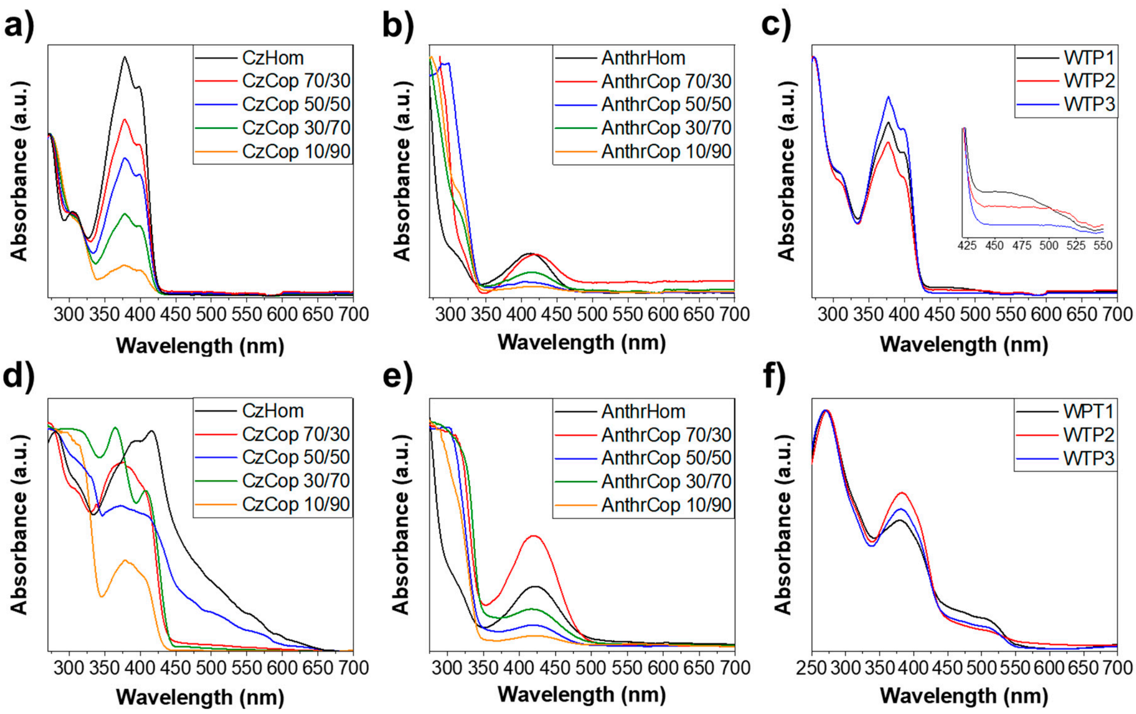

Figure S14. Uv-Vis absorption measurements of **(a)** Carbazole based polymers in DMF solution **(b)** Anthracene based polymers in DMF solution **(c)** White light terpolymers in DMF solution **(d)** Carbazole based polymers in film form **(e)** Anthracene based polymers in film form and **(f)** White light terpolymers in film form. All spectra have been normalized at 375 nm.

Table S4. The electrochemical data of the polymers studied obtained through cyclic voltammetry and absorption spectroscopy.

Code	E_{ox}[a]	λ_{edge}[b]	HOMO	LUMO	Bandgap
**CzHom**	1.57	445	-6.21	-3.42	2.79
**CzCop 10/90**	1.12	432	-5.76	-2.89	2.87
**CzCop 30/70**	1.09	442	-5.59	-2.78	2.81
**CzCop 50/50**	1.14	435	-5.78	-2.93	2.85
**CzCop 70/30**	1.43	440	-6.07	-3.25	2.82
**AnthrHom**	1.06	484	-5.7	-3.14	2.56
**AnthrCop 10/90**	1.07	486	-5.71	-3.16	2.55
**AnthrCop 30/70**	1.22	487	-5.86	-3.31	2.55
**AnthrCop 50/50**	1.14	482	-5.78	-3.21	2.57
**AnthrCop 70/30**	1.14	483	-5.78	-3.21	2.57
**WTP1**	1.35	534	-5.99	-3.67	2.32
**WTP2**	1.33	561	-5.97	-3.76	2.21
**WTP3**	1.58	544	-6.22	-3.94	2.28

a) Determined from the oxidation wave

b) Determined from the absorption spectra of the thin films

f) Electroluminescence Characterization

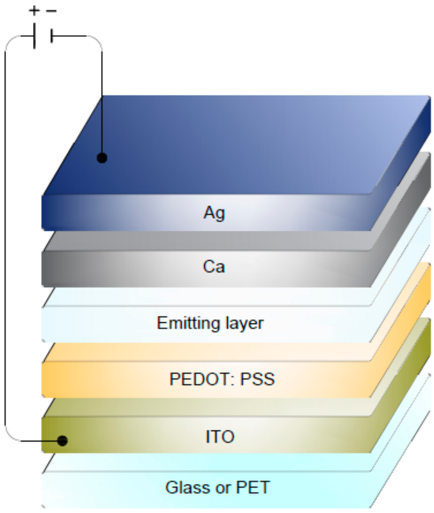

Figure S15. Architecture of the fabricated PLED devices on glass substrate or on flexible PET/ITO substrate.

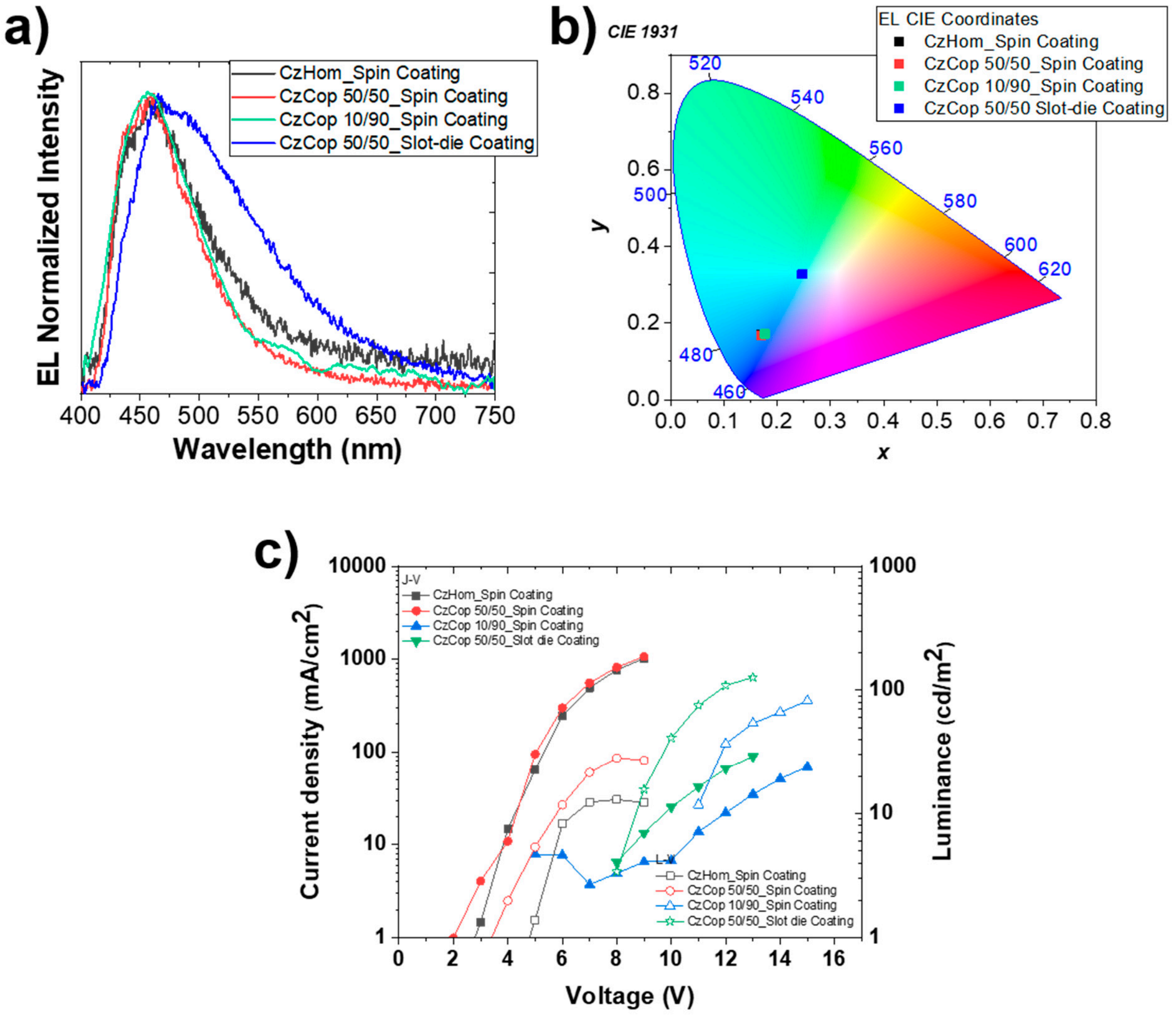

Figure S16. (a) EL spectra of **CzHom**, **CzCop 50/50_Spin coating**, **CzCop 10/90_Spin coating** and **CzCop 50/50_Slot-die coating**, (b) CIE diagram of the EL emission of the studied blue emitting films and (c) characteristics curves of J-V and L-V during the device operation.

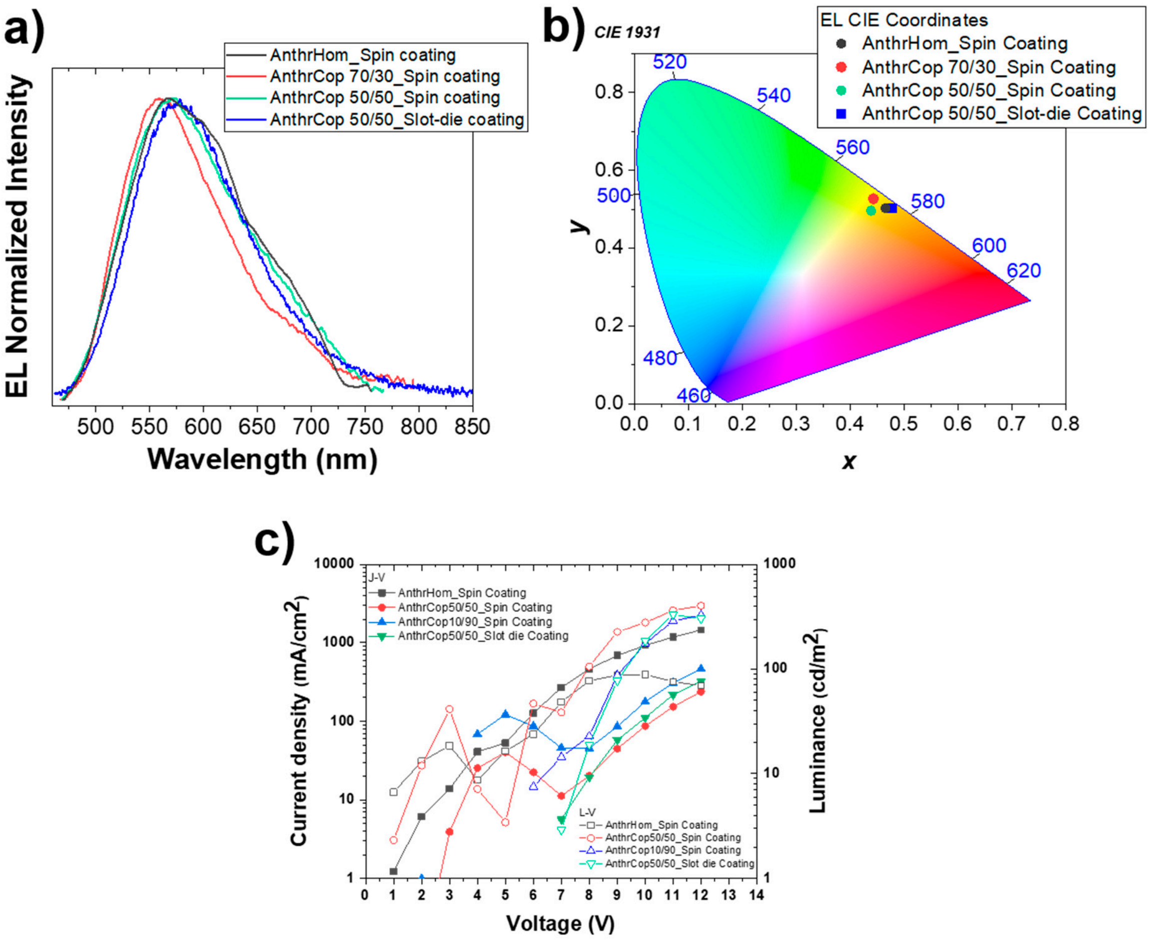

Figure S17. EL spectra of (a) **AnthrHom**, **AnthrCop 70/30**, **AnthrCop 50/50_Spin coating** and **AnthrCop 50/50_Slot-die coating;** (b) CIE diagram of EL yellow emission of the studied **Anthr** based polymers, (c) characteristics curves of J-V and L-V during the device operation.

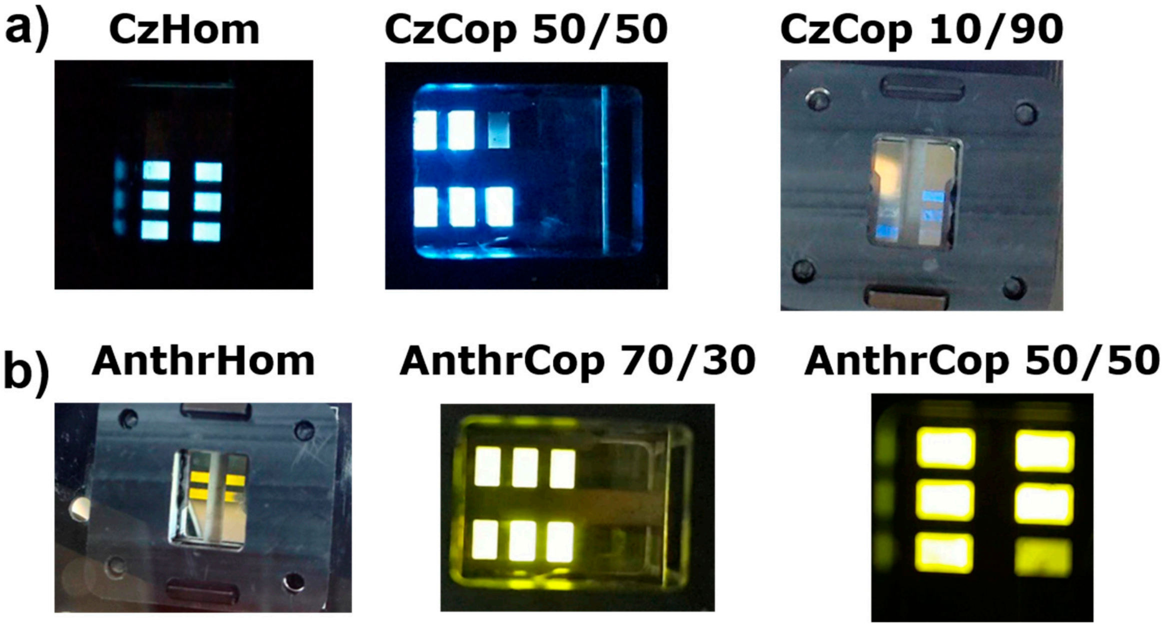

Figure S18. Representative PLED devices on glass substrate of a) Cz based polymers and b) Anthr based polymers.
